# Supplementary material for: Deletion of wfs1 Impairs Oligodendrocyte Precursor Cells Dorsal Distribution and Myelination Through the wfs1-hmgcs1 Axis in Zebrafish
Source: Biology (Basel). 2026 Aug 16;15(16):1402. doi: 10.3390/biology15161402 (PMC13510151; doi:10.3390/biology15161402)
Supplement: Supplementary file 1 [file biology-15-01402-s001.zip › File S1-Transcriptome sequencing data and differentially expressed genes (DEGs) in wfs1-- zebrafish at 5 dpf/1.GO/wfs1KOvsWT/GSEA/wfs1KO/COFACTOR_TRANSPORT/COFACTOR_TRANSPORT(GO_0051181).html]

Details for gene set COFACTOR\_TRANSPORT(GO:0051181)[GSEA]


|  || Dataset | deg.sample |
| Phenotype | sample.cls |
| Upregulated in class | wfs1KO |
| GeneSet | COFACTOR\_TRANSPORT(GO:0051181) |
| Enrichment Score (ES) | 0.53632134 |
| Normalized Enrichment Score (NES) | 1.8143622 |
| Nominal p-value | 0.0 |
| FDR q-value | 0.11359575 |
| FWER p-Value | 0.62 |
Table: GSEA Results Summary

  

Fig 1: Enrichment plot: COFACTOR\_TRANSPORT(GO:0051181)
  
Profile of the Running ES Score & Positions of GeneSet Members on the Rank Ordered List

  

| PROBE | DESCRIPTION   (from dataset) | GENE SYMBOL | GENE\_TITLE | RANK IN GENE LIST | RANK METRIC SCORE | RUNNING ES | CORE ENRICHMENT || 1 | 100006442 | slc25a47b |  |  | 858 | 2.306 | 0.1032 | Yes |
| 2 | 796312 | slc5a6b |  |  | 893 | 2.238 | 0.2284 | Yes |
| 3 | 569329 | flvcr2b |  |  | 1212 | 1.886 | 0.3248 | Yes |
| 4 | 327588 | hpx |  |  | 1254 | 1.848 | 0.4277 | Yes |
| 5 | 407646 | tcn2 |  |  | 2035 | 1.355 | 0.4796 | Yes |
| 6 | 559435 | slc23a1 |  |  | 2401 | 1.208 | 0.5363 | Yes |
| 7 | 564067 | abcb6a |  |  | 5492 | 0.635 | 0.4752 | No |
| 8 | 393385 | slc44a4 |  |  | 6303 | 0.556 | 0.4811 | No |
| 9 | 569608 | slc25a29 |  |  | 10666 | 0.262 | 0.3590 | No |
| 10 | 724009 | slc25a47a |  |  | 11921 | 0.195 | 0.3306 | No |
| 11 | 436640 | slc25a48 |  |  | 12371 | 0.170 | 0.3262 | No |
| 12 | 792763 | slc48a1b |  |  | 12551 | 0.162 | 0.3297 | No |
| 13 | 100002010 | abcc1 |  |  | 14081 | 0.088 | 0.2867 | No |
| 14 | 403024 | slc25a19 |  |  | 14161 | 0.084 | 0.2889 | No |
| 15 | 563356 | slc5a6a |  |  | 14502 | 0.069 | 0.2822 | No |
| 16 | 497434 | slc19a1 |  |  | 14813 | 0.055 | 0.2755 | No |
| 17 | 100004801 | abcb6b |  |  | 15866 | 0.004 | 0.2427 | No |
| 18 | 566658 | si:ch211-117m20.5 |  |  | 15991 | -0.001 | 0.2389 | No |
| 19 | 751743 | slc25a42 |  |  | 18250 | -0.087 | 0.1730 | No |
| 20 | 565210 | flvcr2a |  |  | 18312 | -0.090 | 0.1761 | No |
| 21 | 336147 | abcc5 |  |  | 20324 | -0.180 | 0.1232 | No |
| 22 | 562595 | flvcr1 |  |  | 21403 | -0.236 | 0.1027 | No |
| 23 | 436697 | slc48a1a |  |  | 24205 | -0.407 | 0.0377 | No |
| 24 | 566714 | tcnl |  |  | 28811 | -0.880 | -0.0572 | No |
| 25 | 100321249 | slc23a2 |  |  | 30124 | -1.301 | -0.0250 | No |
| 26 | 560478 | slc25a26 |  |  | 30366 | -1.425 | 0.0478 | No |
Table: GSEA details
[plain text format]

  

Fig 2: COFACTOR\_TRANSPORT(GO:0051181)
  
Blue-Pink O' Gram in the Space of the Analyzed GeneSet

  

Fig 3: COFACTOR\_TRANSPORT(GO:0051181): Random ES distribution
  
Gene set null distribution of ES for
 **COFACTOR\_TRANSPORT(GO:0051181)**
